# TGFβ2 Induces the Formation of Cross-Linked Actin Networks (CLANs) in Human Trabecular Meshwork Cells Through the Smad and Non-Smad Dependent Pathways

**DOI:** 10.1167/iovs.16-19672

**Published:** 2017-02

**Authors:** Michela Montecchi-Palmer, Jaclyn Y. Bermudez, Hannah C. Webber, Gaurang C. Patel, Abbot F. Clark, Weiming Mao

**Affiliations:** North Texas Eye Research Institute, University of North Texas Health Science Center, Fort Worth, Texas, United States

**Keywords:** glaucoma, trabecular meshwork, TGFβ2, cross-linked actin networks

## Abstract

**Purpose:**

Increased intraocular pressure results from increased aqueous humor (AH) outflow resistance at the trabecular meshwork (TM) due to pathologic changes including the formation of cross-linked actin networks (CLANs). Transforming growth factor β2 (TGFβ2) is elevated in the AH and TM of primary open angle glaucoma (POAG) patients and induces POAG-associated TM changes, including CLANs. We determined the role of individual TGFβ2 signaling pathways in CLAN formation.

**Methods:**

Cultured nonglaucomatous human TM (NTM) cells were treated with control or TGFβ2, with or without the inhibitors of TGFβ receptor, Smad3, c-Jun N-terminal kinases (JNK), extracellular signal regulated kinase (ERK), P38, or Rho-associated protein kinase (ROCK). NTM cells were cotreated with TGFβ2 plus inhibitors for 10 days or pretreated with TGFβ2 for 10 days followed by 1-hour inhibitor treatment. NTM cells were immunostained with phalloidin-Alexa-488 and 4′,6-diamidino-2-phenylindole (DAPI). Data were analyzed using 1-way ANOVA and Dunnett's post hoc test.

**Results:**

TGFβ2 significantly induced CLAN formation (*n* = 6 to 12, *P* < 0.05), which was completely inhibited by TGFβ receptor, Smad3, and ERK inhibitors, as well as completely or partially inhibited by JNK, P38, and ROCK inhibitors, depending on cell strains. One-hour exposure to ROCK inhibitor completely resolved formed CLANs (*P* < 0.05), whereas TGFβ receptor, Smad3 inhibitor, and ERK inhibitors resulted in partial or complete resolution. The JNK and P38 inhibitors showed partial or no resolution. Among these inhibitors, the ROCK inhibitor was the most disruptive to the actin stress fibers, whereas ERK inhibition showed the least disruption.

**Conclusions:**

TGFβ2-induced CLANs in NTM cells were prevented and resolved using various pathway inhibitors. Apart from CLAN inhibition, some of these inhibitors also had different effects on actin stress fibers.

Glaucoma is a progressive optic neuropathy affecting both the anterior and posterior segments of the eye and is a leading cause of irreversible vision loss and blindness worldwide. It is estimated that glaucoma will affect more than 80 million people by the year 2020.^1–3^ The most common form of glaucoma is primary open-angle glaucoma (POAG), which is characterized by painless, progressive, and irreversible vision loss. Although the exact disease mechanism(s) of POAG is not fully understood, elevated intraocular pressure (IOP) is the primary risk factor for the development and progression of POAG. In these patients, IOP elevation is caused by increased aqueous humor (AH) outflow resistance at the trabecular meshwork (TM). This increased resistance has been associated with loss of TM cells, excessive extracellular matrix (ECM) accumulation, and cytoskeletal reorganization.^4–11^

We are particularly interested in the actin cytoskeletal reorganization in TM cells. We first reported the formation of cross-linked actin networks (CLANs) in both human trabecular meshwork (HTM) cells and tissues.^4,8,12^ CLANs are three-dimensional, geodesic dome-like structures formed primarily around the nucleus, although they can also be found throughout the cell. In two-dimensional microscopic views, CLANs appear to be web-like structures and composed of numerous “hubs and spokes.” Although cultured cell types other than TM cells form transient CLAN-like structures during the process of cell attachment and spreading,^13–17^ only TM cells form and retain CLANs when they are confluent.^12^

In cultured TM cells, CLAN formation can be induced by glucocorticoids and by transforming growth factor β2 (TGFβ2). TGFβ2 is elevated in the AH and TM of POAG patients and induces ocular hypertension in perfusion cultured human and porcine anterior segments, as well as in mouse eyes.^9,18–23^ In cultured TM cells, TGFβ2 induces the expression of ECM proteins such as fibronectin (FN) and factors that suppress proteolytic degradation of the ECM.^24–26^ Additionally, TGFβ2 increases the expression of ECM cross-linking enzymes such as lysyl oxidase (LOX)^27^ and transglutaminase-2.^28,29^

We previously identified an association between CLANs and POAG by comparing glaucomatous human TM (GTM) cells to nonglaucomatous TM (NTM) cells.^5,30^ In those studies, we observed that approximately 40% of the GTM cells contain CLANs, whereas only 4% of the NTM cells have CLANs. Specific mathematical models predict that CLANs increase the stiffness of actin filaments by two orders of magnitude.^31^ Other studies showed that GTM tissues are stiffer compared with NTM tissues.^32,33^ Although those studies did not evaluate the direct correlation between CLAN formation and TM stiffness, it is believed that CLANs increase AH outflow resistance and IOP by increasing TM stiffness, as well as disturbing TM homeostasis.^12^

TGFβ2 signaling has two primary pathways for signal transduction: the Smad-dependent (Smad pathway) and -independent (non-Smad pathway) pathways. Both pathways are activated by TGFβ2 binding to the TGFβ receptor complex, which is then autophosphorylated. In the Smad pathway, the activated receptor complex phosphorylates Smad2 and Smad3, which then binds with Smad4 prior to translocation to the nucleus. The non-Smad pathway includes various branches of mitogen-activated protein kinase (MAPK) pathways such as extracellular signal regulated kinase (ERK), c-Jun N-terminal kinases (JNK), and P38 kinases, as well as Rho-like guanosine triphosphatase (GTPase) signaling pathways.^34^

Rho-associated protein kinase (ROCK) inhibitors have the ability to reduce trabecular outflow resistance and IOP.^35–40^ The Rho/ROCK signaling pathway acts as a molecular switch in the regulation of focal adhesions, cellular contraction, cellular motility, cytokinesis, and the formation of actin stress fibers.^41^ It is believed that the ocular hypotensive effect of ROCK inhibitors is due to “relaxation” of the TM cytoskeleton.^37,41–43^ Inhibition of ROCK decreases actin polymerization, relaxes the TM cells, and decreases outflow resistance. However, whether ROCK inhibition affects CLAN formation is not clear.

Because of the complexity of the TGFβ signaling, the delineation of which exact pathway is responsible for a particular biological response is difficult to predict. In the present study, we used NTM cells and various pathway inhibitors to determine the role of individual TGFβ signaling pathways in the formation and stabilization of CLANs.

## Methods

### NTM Cell Cultures

Two primary NTM cell strains (NTM1022-02, and NTM30A) were used for these experiments and were generated and maintained as previously published.^4,5,12^ Cells were plated onto 12-mm glass coverslips and grown to 100% confluency in low glucose Dulbecco's modified Eagle's medium (DMEM; Invitrogen-Gibco Life Technologies, Grand Island, NY, USA) containing 10% to 20% fetal bovine serum (FBS; Atlas Biologicals Products, Fort Collins, CO, USA) and penicillin (100 U/mL), streptomycin (0.1 mg/mL), and l-glutamine (0.292 mg/mL; Invitrogen-Gibco Life Technologies).

### Cell Treatments

Treatments were administered in high glucose DMEM with 1% FBS. High glucose medium was used to improve cell survival, and a low concentration of FBS minimized confounding effects of endogenous growth factors that may be present in the serum. Confluent NTM cells were treated with TGFβ2 (5 ng/mL) (RD Systems, Minneapolis, MN, USA) to induce CLAN formation or DMEM with or without dimethyl sulfoxide (DMSO; vehicle control for MAPK inhibitors). To inhibit the Smad and non-Smad pathways, NTM cells were cotreated with TGFβ2 and TGFβ receptor type I inhibitor SB431542 (5 μM; Sigma, Saint Louis, MO, USA).^27^ To inhibit the Smad pathway, NTM cells were cotreated with TGFβ2 and the Smad3 phosphorylation inhibitor SIS3 (10 μM; Sigma).^27^ To inhibit the non-Smad pathways, NTM cells were cotreated with TGFβ2 and the JNK inhibitor SP600125 (10 μM; CalBioChem, San Diego, CA, USA),^27^ MEK/Erk inhibitor U0126 (25 μM; Promega, Maddison, WI, USA),^44^ P38 inhibitor SB203580 (5μM; Tocris BioSci, Ellisville, MO, USA),^27^ or ROCK inhibitor Y27632 (10 μM; Sigma).^35,37,44,45^ All these inhibitors were used in previous TM studies and have demonstrated successful pathway inhibition at the concentrations described previously. Cells were cotreated with TGFβ2 together with pathway inhibitors for 10 days for studying the prevention of CLAN formation or pretreated with TGFβ2 for 10 days followed by treatment with individual inhibitors for 1 hour to study CLAN resolution. Each treatment group consisted of 6 to 12 coverslips (*n* = 6 to 12). Medium was changed every 2 to 3 days.

### Epifluorescent Staining of CLANs

NTM cells were fixed with 2% paraformaldehyde in PBS, washed with PBS, permeabilized using 0.5% Triton X-100, and blocked with Superblock (Thermo Scientific, Waltham, MA, USA). F-actin was stained with Phalloidin conjugated with Alexa-488 (1:100; Life Technologies, Eugene, OR, USA) for 1 hour at room temperature. After PBS washes, coverslips were mounted onto slides using ProLong Gold Anti-Fade with 4′,6-diamidino-2-phenylindole (DAPI; Life Technologies) for nuclear counterstaining.

### Evaluation of CLANs

CLANs were visualized using the Nikon Eclipse Ti inverted fluorescence microscope (Nikon, Inc., Melville, NY, USA) with 600× magnification. Cytoskeletal images were taken using the Nikon Eclipse Ti inverted fluorescence microscope equipped with the Cri Nuance FX Camera System (Perkin-Elmer, Inc., Waltham, MA, USA).

**Figure 1:**
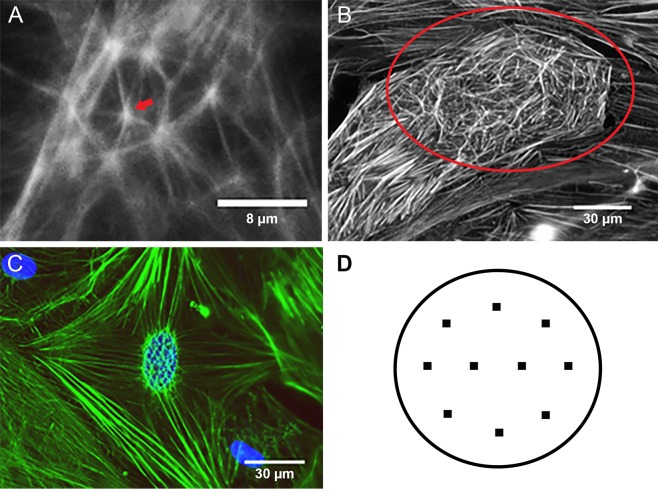
Morphology and evaluation of CLANs. (**A**) Representative image of a single CLAN in an NTM cell. The CLANs consist of distinct hubs (*red arrow*) and F-actin spokes, forming a dome-like structure. A minimum of three hubs creating at least one triangulated actin arrangement can be counted as a CLAN. (**B**) Representative image of multiple CLANs within a single cell (600× magnification). (**C**) Representative image of multiple CLANs at the perinuclear region (600× magnification). *Blue*: DAPI. (**D**) CLAN formation (percentage of CPCs) was evaluated in 10 representative areas (dots) of each coverslip.

CLANs were defined as F-actin–containing cytoskeletal structures with at least one triangulated actin arrangement consisting of actin spokes and at least three identifiable hubs.^46^ Representative images of CLANs are shown in Figures 1A–1C. Each coverslip was assessed at 10 locations (Fig. 1D) with approximately 100 to 150 cells per coverslip. Six to 12 coverslips were evaluated per treatment group.

All CLAN counting was done in a masked manner. CLAN-positive cells (CPCs) were defined as any cell containing at least one CLAN or multiple CLANs. The formation of CLANs was compared using the percentage of CPCs, which is calculated by dividing the number of CPCs by the number of DAPI-positive cells.

### Western Immunoblotting

Primary NTM cells were cultured in six-well plates as previously described. When cells were confluent, they were treated with 5 ng/mL TGFβ2 with or without the TGFβ pathway inhibitors described previously for 24 hours. After treatment, whole cell lysates were collected using M-PER buffer (Thermo Scientific) or two-dimensional electrophoresis sample rehydration buffer. After protein estimation using the DC protein assay kit (Bio-Rad, Hercules, CA, USA) or the EZQ kit (Thermo Scientific), 38 μg total protein was used for SDS-PAGE. After electrophoresis, proteins were transferred onto a polyvinylidene fluoride membrane. The membrane was blocked with Odyssey blocking buffer (Li-Cor, Licoln, NE, USA), incubated with one of the following antibodies: rabbit polyclonal anti–α-smooth muscle actin antibody (1:1000; Abcam, Cambridge, MA, USA); rabbit anti-JNK (1:1000; Cell Signaling Technology); rabbit anti-pJNK (1:1000; Cell Signaling Technology); rabbit anti-ERK (1:1000; Cell Signaling Technology); rabbit anti-pERK (1:1000; Cell Signaling Technology); or mouse anti-β-actin (1:2000; Millipore, Billerica, MA, USA).

After incubation, the membrane was washed, incubated with a secondary antibody (goat anti-rabbit or goat anti-mouse) conjugated with horseradish peroxidase (Cell Signaling Technology). Signals were developed using the SuperSignal West Femto Maximum Sensitivity Substrate (Thermo Scientific). Images were taken using Odyssey Fc Dual-Mode imaging system (Li-Cor).

### Statistical Analysis

The percentage of CPCs was compared using 1-way ANOVA followed by Dunnett's multiple comparisons post hoc test (GraphPad Prism 6.02; GraphPad Software, Inc., La Jolla, CA, USA). Data are presented as mean ± SEM, with the significance level set at *P* < 0.05.

## Results

### Smad and Non-Smad Pathway Inhibitors Prevented CLAN Formation

We first studied whether inhibition of Smad and/or non-Smad pathways would inhibit CLAN formation. We treated human NTM cells with TGFβ2 together with inhibitors against the TGFβ pathways (SB431542), the Smad pathway (SIS3), the ERK pathway (U0126), the JNK pathway (SP600125), the P38 pathway (SB203580), or the ROCK pathway (Y27632). Because CLAN formation has been shown to peak after 10 to 14 days of TGFβ2 exposure,^47^ we treated NTM cells for 10 days to ensure CLAN induction. Data are presented as the percentage of CPCs.

**Figure 2:**
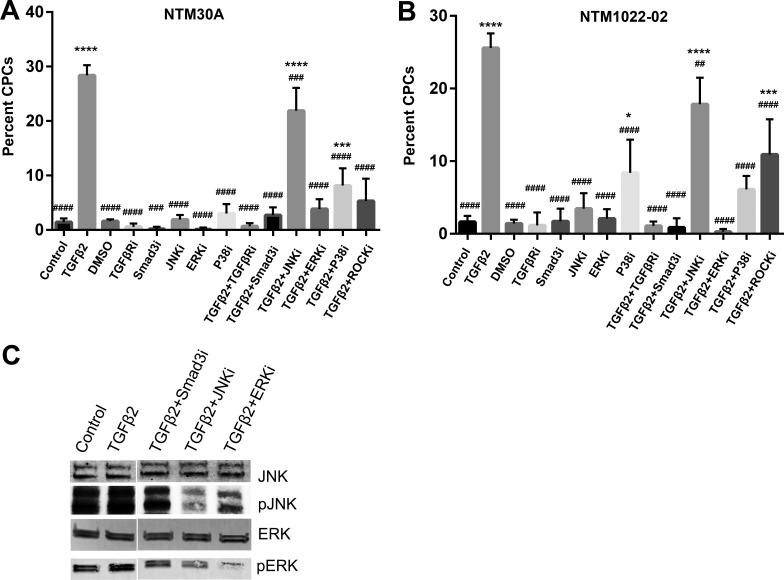
Prevention of CLAN formation in NTM cells by TGFβ pathway inhibitors. (**A**) NTM30A and (**B**) NTM1022-02 cells cultured on glass coverslips (*n* = 6 to 12) were treated with control or TGFβ2 with or without indicated TGFβ Smad or non-Smad pathway inhibitors for 10 days. Percentage of CPCs was compared using 1-way ANOVA with Dunnett's multiple comparisons post hoc test. *Columns and bars*: means and SEM. *P < 0.05 for the group of interest versus control; ***P < 0.001, ****P < 0.0001, and ^##^*P* < 0.01 for the group of interest versus TGFβ2; ^###^*P* < 0.001; ^####^*P* < 0.0001. TGFβRi, TGFβ receptor inhibitor (SB431542; 5 μM); SMAD3i, Smad3 phosphorylation inhibitor (SIS3; 10 μM); JNKi, JNK pathway inhibitor (SP600125; 10 μM); ERKi, ERK pathway inhibitor (U0126; 25 μM); P38i, P38 pathway inhibitor (SB203580; 5 μM); ROCKi, ROCK pathway inhibitor (Y27632; 10 μM). (**C**) NTM1022-02 cells were treated with TGFβ2 with or without indicated inhibitors, and whole cell lysates were collected for WB. pJNK, phosphorylated JNK; pERK, phosphorylated ERK.

In NTM30A cells receiving vehicle controls (medium alone or medium with DMSO), the percentage of CPCs was 1.44 ± 0.19% (SEM) and 1.62 ± 0.14%, respectively (Fig. 2A). These data are similar to our previous reports.^5^ In contrast, TGFβ2-treated TM cells had 28.40 ± 1.87% CPCs (*P* < 0.0001 versus controls), confirming that TGFβ2 is a potent CLAN inducer.

Cotreatment with TGFβ2 and TGFβ receptor inhibitor (SB431542) or inhibitor of the Smad signaling pathway (SIS3) decreased the percentage of CPCs to 0.68 ± 0.24% and 2.7 ± 0.65%, respectively (*P* < 0.0001 versus TGFβ2), showing their complete inhibition of TGFβ2-induced CLAN formation (Fig. 2A).

Different from the Smad pathway, inhibition of the non-Smad pathway had different effects on CLAN formation (Fig. 2A). The ERK pathway inhibitor (U0126) and ROCK pathway inhibitor (Y27632) resulted in 3.84 ± 0.74% and 5.33 ± 1.66% CPCs (*P* < 0.0001 versus TGFβ2), respectively, which demonstrated a complete inhibition of TGFβ2-induced CLAN formation. However, the JNK pathway inhibitor (SP600125) and P38 pathway inhibitor (SB203580) resulted in only partial inhibition of CLAN formation, with 21.90 ± 1.72% and 8.17 ± 1.29% CPCs (*P* < 0.001 versus TGFβ2 and *P* < 0.001 versus control). Treatment with inhibitors alone did not significantly change CLAN formation at the basal level (*P* > 0.05; Fig. 2A).

The formation of CLANs was mostly consistent in the two NTM cell strains studied. The exceptions in NTM1022-02 cells are (1) the ROCK pathway inhibitor showed partial inhibition of CLAN formation and (2) the P38 pathway inhibitor alone slightly elevated CLAN formation (*P* < 0.05 versus control; Fig. 2B). These data suggest that the response of TM cells to individual pathway inhibitors may be strain dependent.

Because there is a difference in CLAN prevention between JNK and ERK inhibitors, we studied their efficacy in inhibition of JNK and ERK phosphorylation using Western blotting (WB) as a validation. We found that both JNK and ERK inhibitors blocked the phosphorylation of JNK and ERK, respectively (Fig. 2C).

### TGFβ Pathway Inhibitors Resolved Already Formed CLANs

Although we found that TGFβ pathway inhibitors prevented TGFβ2-induced CLAN formation, it is still unclear whether they can also resolve already formed CLANs. Therefore, we pretreated NTM cells with TGFβ2 for 10 days to induce CLANs and then treated them with individual inhibitors for 1 hour. Cotreatment of TGFβ2 and those inhibitors for 10 days was also incorporated as a positive control.

**Figure 3:**
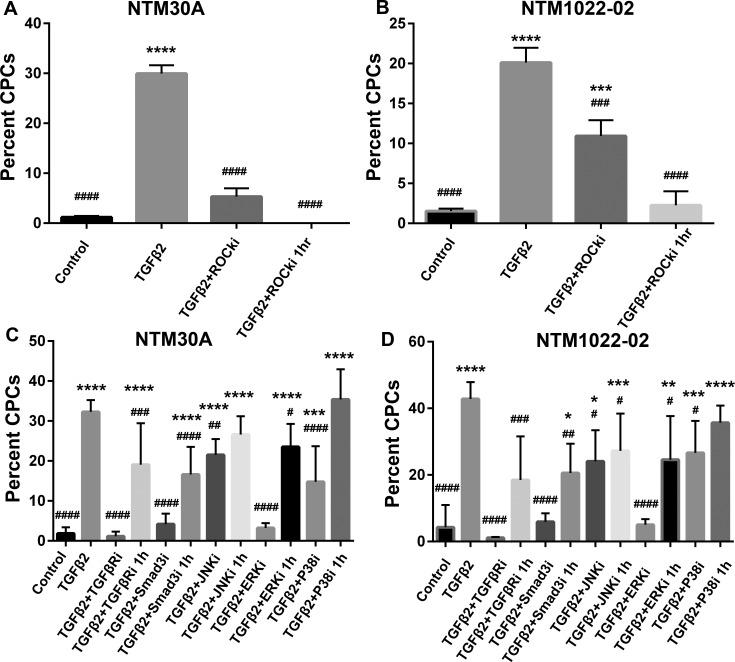
Resolution of CLAN formation in NTM cells by TGFβ pathway inhibitors. NTM cells cultured on glass coverslips (*n* = 6) were pretreated with TGFβ2 for 10 days, followed by 1-hour treatment of the indicated inhibitors. Some cells were also cotreated with TGFβ2 and the indicated inhibitor for 10 days as a positive control. Percentage of CPCs was compared using 1-way ANOVA with Dunnett's multiple comparisons post hoc test. (**A**, **B**) NTM cells treated with the ROCK inhibitor. (**C**, **D**) NTM cells treated with the other inhibitors. *Columns and bars*: means and SEM. *P < 0.05 for group of interest versus control; **P < 0.01, ***P < 0.001, ****P < 0.0001, and ^#^*P* < 0.05 for the group of interest versus TGFβ2; ^##^*P* < 0.01, ^###^*P* < 0.001, and ^####^*P* < 0.0001. TGFβRi, TGFβ receptor inhibitor (SB431542; 5 μM); SMAD3i, Smad3 phosphorylation inhibitor (SIS3; 10 μM); JNKi, JNK pathway inhibitor (SP600125; 10 μM); ERKi, ERK pathway inhibitor (U0126; 25 μM); P38i, P38 pathway inhibitor (SB203580; 5 μM); ROCKi, ROCK pathway inhibitor (Y27632; 10 μM).

In both TM cell strains, we found that 1-hour treatment with the ROCK inhibitor completely removed already formed CLANs, resulting in 0.00 ± 0.00% (NTM30A) and 2.25 ± 0.72% (NTM1022-02) CPCs (*P* < 0.0001 versus TGFβ2; Figs. 3A, 3B).

In contrast, the effects of the inhibition of non-ROCK pathways were more diverse. In NTM30A cells, the TGFβ receptor inhibitor (SB431542), Smad3 inhibitor (SIS3), and ERK inhibitor (U0126) partially resolved preformed CLANs, with 19.05 ± 4.64%, 16.61 ± 2.83%, and 23.51 ± 2.36% CPCs (all *P* < 0.05 versus TGFβ2 and *P* < 0.0001 versus control), respectively (Fig. 3C). However, JNK or P38 inhibition did not show significant resolution of formed CLANs (*P* > 0.05 versus TGFβ2). The results from NTM1022-02 cells are similar, except that the TGFβ receptor inhibitor showed complete inhibition and the JNK inhibitor showed partial inhibition (Fig. 3D).

### Differential Effects of Smad and Non-Smad Pathway Inhibition on Actin Stress Fiber Formation

It is unclear whether the TGFβ pathway regulates CLANs and actin stress fibers in the same manner. Therefore, we evaluated the effects of individual TGFβ pathway inhibitors on actin stress fibers in TM cells. It is our belief that a compound that is able to prevent/remove CLANs but has minimal impact on normal actin cytoskeleton structures such as stress fibers will treat one of the glaucomatous pathologies (CLANs), while potentially preserving/restoring the normal actin cytoskeleton.

**Figure 4:**
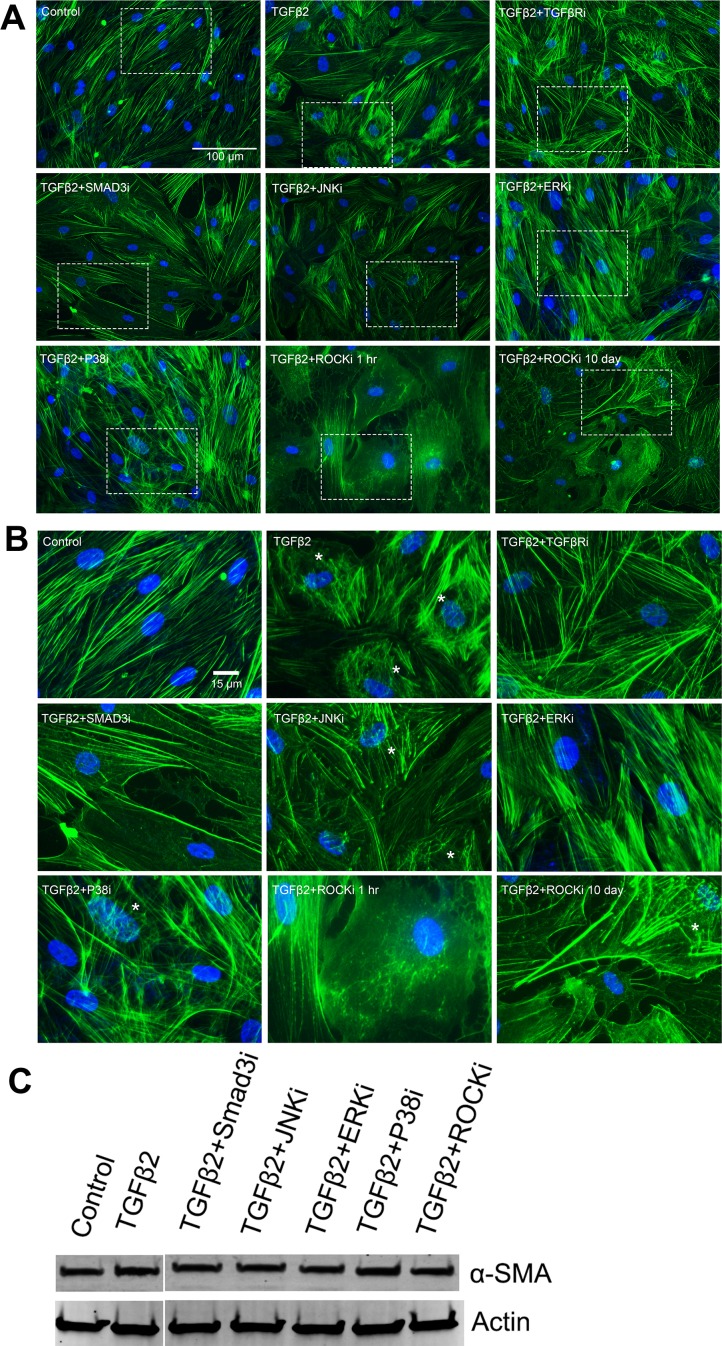
The effects of TGFβ pathway inhibitors on stress fiber formation in NTM cells. NTM cells cultured on glass coverslips (*n* = 6) were cotreated with TGFβ2 and the indicated inhibitors for 10 days. Some cells were treated with TGFβ2 for 10 days, followed by 1-hour ROCK inhibitor treatment. Cells were fixed and used for immunofluorescent staining. (**A**) Representative images of actin stress fibers with inhibitor treatment. (**B**) Enlarged images from corresponding areas in **A**. Asterisks, CLANs; TGFβRi, TGFβ receptor inhibitor (SB431542; 5 μM); Smad3i, Smad3 phosphorylation inhibitor (SIS3; 10 μM); JNKi, JNK pathway inhibitor (SP600125; 10 μM); ERKi, ERK pathway inhibitor (U0126; 25 μM); P38i, P38 pathway inhibitor (SB203580; 5 μM); ROCKi, ROCK pathway inhibitor (Y27632; 10 μM). (**C**) NTM cells were treated with TGFβ2 with or without indicated inhibitors, and whole cell lysates were collected for WB to study α-SMA.

As previously reported,^44^ TGFβ2 increased and reorganized actin stress fiber formation compared to control (Figs. 4A, 4B). Transforming growth factor β receptor and Smad3 inhibitors restored actin stress fibers to baseline levels (versus control). The JNK or P38 inhibitors had little effect on TGFβ2-induced stress fibers. The ERK inhibitor, although it did not block TGFβ2-induced stress fibers, seemed to preserve the normal arrangement of those fibers. Different from these other inhibitors, 1-hour treatment with the ROCK inhibitor almost completely eliminated stress fibers. However, some stress fibers reformed after 10-day cotreatment with TGFβ2 plus ROCK inhibitor.

Because α-smooth muscle actin (α-SMA) is a key component of CLANs ^12^ and is closely associated with actin, we studied whether kinase inhibitors affect TGFβ2-induced α-SMA expression in HTM cells using WB. We did not observe obvious inhibition of α-SMA expression (Fig. 4C).

## Discussion

In this study, we found that TGFβ2-induced CLANs in NTM cells could be both prevented and resolved using different TGFβ pathway inhibitors. These inhibitors had different effects on actin stress fibers, which did not completely match their effects on CLANs.

**Figure 5:**
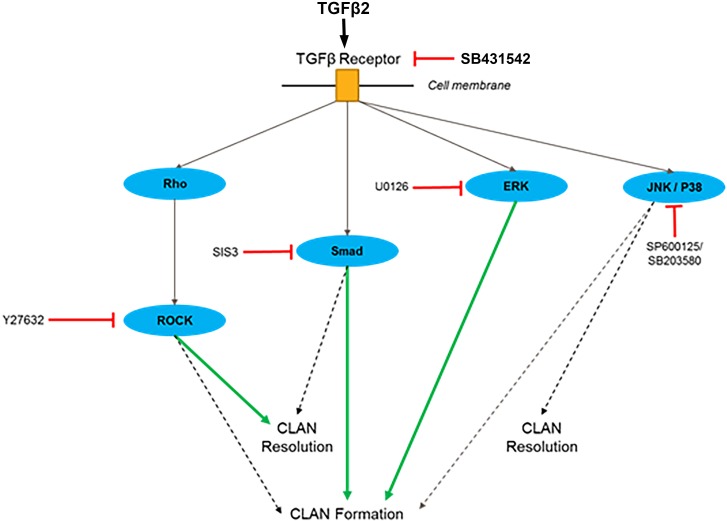
Hypothesized roles of the TGFβ2 pathway and inhibitors in CLAN formation. *Solid green lines*, pathways that may play major roles in CLAN formation or maintenance; *dotted lines*, pathways that may play minor roles in CLAN formation or maintenance.

Our results suggest that TGFβ2 induced CLAN formation occurs via both the Smad and non-Smad TGFβ pathways (summarized in Fig. 5). However, the contribution of individual TGFβ2 signaling pathways to CLAN formation seems to be different. In the two NTM cell strains studied, both the Smad pathway and non-Smad ERK pathways seemed to play major roles in TGFβ2-induced CLAN formation. The JNK and P38 pathway only partially participated in CLAN induction, whereas the contribution of the ROCK pathway was cell strain dependent. However, the overall trend was consistent.

O'Reilly et al. previously showed that, in bovine TM cells, the TGFβ receptor inhibitors LY-364997 and SB431542 partially inhibited TGFβ2-induced CLAN formation,^47^ which is different from our findings where we found total inhibition. However, the Smad3 inhibitor SIS3 completely inhibited CLAN formation, which is consistent with our observations in NTM cells. The difference between the two studies may be due to the difference in species, suggesting that for results generated in nonhuman TM cells/tissues, a confirmation in human TM is necessary.

We also studied whether different TGFβ pathway inhibitors could remove already formed CLANs, which to our best knowledge, has not been previously determined. It surprised us that only 1-hour treatment with TGFβ receptor inhibitor, Smad inhibitor, ERK inhibitor, and ROCK inhibitor completely or partially removed already formed CLANs. Because 1 hour is generally believed to be too short to affect both transcription and translation, these changes are very likely to be due to alterations in protein modification, especially protein phosphorylation. Alternatively, CLAN formation may be a more dynamic process than initially thought, and TGFβ signaling is required to maintain CLANs. Therefore, even a short-term disruption of the TGFβ pathway is able to disrupt CLANs.

Whether the formation of CLANs and actin stress fibers share a similar mechanism is not clear. Our findings suggest that each TGFβ signaling pathway had different impacts on actin stress fiber formation, which was different from their impact on CLANs. Although it is known that relaxation of the actin cytoskeleton (disassembly of actin stress fibers) is a valid approach to lower IOP (as seen with ROCK inhibitors), it still remains unknown if long-term disruption of normal actin stress fibers will impair TM function. Also, the most common side effect with the use of ROCK inhibitors is ocular hyperemia because they also “relax” conjunctival blood vessels similar to their effect on the TM. Therefore, if a compound is able to remove CLANs with minimal or no changes in actin stress fibers, it may have ocular hypotensive effects with less side effects such as hyperemia.

This is our first attempt to dissect the role of individual TGFβ signaling pathways in CLAN formation. There are still many unanswered questions.

First, it is still unclear whether these individual pathways work independently or in a synergistic manner. We found that inhibition of either the Smad or Non-Smad pathway was able to completely inhibit CLAN formation. Therefore, we believe that these pathways are more likely to work synergistically. If they do work together, it will be important to determine at which molecules this cross-regulation occurs.

Second, the downstream molecules of the TGFβ pathway that mediate CLAN formation are not entirely clear. Peters et al. performed a proteomic study using spreading TM cells.^48^ The authors first treated confluent TM cells with dexamethasone (DEX) to induce CLAN formation or with ethanol as a vehicle control. Then they dissociated and seeded TM cells into fibronectin coated dishes. The TM proteins were fractionated and compared using mass spectrometry. They observed a change in 318 cytoskeletal proteins, some of which contained phosphorylated residues suggesting DEX affects the expression of these proteins at the transcriptional and translational levels. That study provided insightful information of CLAN-related proteins. However, many adherent cell types form transient CLANs when they are first attaching and spreading, whereas only TM cells form and retain CLANs when they are confluent. Therefore, it is still unclear whether the CLANs formed during cell spreading share the same signaling pathway and biological components as the CLANs formed in confluent cells. Also, it is known that TGFβ2 induces mesenchymal-like features in the TM including α-SMA,^44^ which is an important component of CLANs.^12^ We found that TGFβ2-induced α-SMA was not obviously affected by the inhibitors after a 24-hour cotreatment, which is different from a study reported by Padmanabhan et al. in which the authors showed the ROCK inhibitor Y27632 decreased α-SMA after a 2-hour treatment without TGFβ2.^44^ We believe this could result from different treatment times and regimens. A prolonged treatment may lead to desensitization. Also, the presence of TGFβ2 may counteract the effect of Y27632, especially after prolonged treatment because Y27632 is a rapid acting compound. However, our findings do not exclude the role of α-SMA in CLAN formation because our WB data did not provide information of posttranslational modification of α-SMA. Besides α-SMA, myosin light chain kinase (MLCK), a key component of the Rho-Rock pathway and stress fiber formation, may also be an important player in CLAN formation. Although there has been no direct evidence, our recent publication shows that myosin light chain, a downstream molecule and substrate of MLCK, is in close proximity to CLANs.^49^ Further research is required to determine whether MLCK is involved in the initial assembly of CLANs and/or the maintenance of CLANs.

Third, although both glucocorticoids and TGFβ2 are able to induce morphologically similar CLANs via different pathways, it is unknown if TGFβ2-induced CLANs and DEX-induced CLANs are biochemically and/or mechanically identical. If the mechanisms are the same, there must be a set of common mediator molecules shared by the two pathways.

Finally, the role of connective tissue growth factor (CTGF) in CLAN formation has not yet been determined. CTGF is a profibrotic cytokine that mediates TGFβ2 signaling in the TM.^50^ CTGF is among the most highly expressed genes in the TM.^51^ It is elevated in the AH of exfoliation glaucoma patients^50,52^ and plays a critical role in cell migration, adhesion, proliferation, matrix production, and mediates several of the downstream actions of TGFβ.^53^ Overexpression of CTGF in a mouse model induces ocular hypertension and optic nerve damage.^54^ ROCK inhibitors also affect both TGFβ2 and CTGF-induced cellular and cytoskeletal changes in a similar manner.^54,55^ Therefore, more research is needed to determine the effect of CTGF and its inhibitors on CLAN formation.

In summary, we found that different TGFβ signaling pathways play different roles in the inhibition and disassembly of CLANs, as well as in actin stress fiber formation. This information may help in the development of novel ocular hypotensive agents.
